# Machine Learning-Based Tomato Fruit Shape Classification System

**DOI:** 10.3390/plants13172357

**Published:** 2024-08-23

**Authors:** Dana V. Vazquez, Flavio E. Spetale, Amol N. Nankar, Stanislava Grozeva, Gustavo R. Rodríguez

**Affiliations:** 1 Instituto de Investigaciones en Ciencias Agrarias de Rosario, Consejo Nacional de Investigaciones Científicas y Técnicas, Universidad Nacional de Rosario (IICAR-CONICET-UNR), Campo Experimental Villarino, Zavalla S2125ZAA, Argentina; vazquez@iicar-conicet.gob.ar (D.V.V.); rodriguez@iicar-conicet.gob.ar (G.R.R.); 2 Facultad de Ciencias Agrarias, Universidad Nacional de Rosario, Parque Villarino, CC Nº 14, Zavalla S2125ZAA, Argentina; 3 Centro Internacional Franco Argentino de Ciencias de la Información y de Sistemas, Consejo Nacional de Investigaciones Científicas y Técnicas, Universidad Nacional de Rosario (CIFASIS-CONICET-UNR), 27 de Febrero 210 bis, Rosario S2000EZP, Argentina; 4 Horticulture Department, University of Georgia, Tifton, GA 31793, USA; amolnankar@uga.edu; 5 Maritsa Vegetable Crops Research Institute (MVCRI), 4003 Plovdiv, Bulgaria; stanislava_grozeva@abv.bg

**Keywords:** morphology recognition, feature extraction, support vector machine

## Abstract

Fruit shape significantly impacts the quality and commercial value of tomatoes (*Solanum lycopersicum* L.). Precise grading is essential to elucidate the genetic basis of fruit shape in breeding programs, cultivar descriptions, and variety registration. Despite this, fruit shape classification is still primarily based on subjective visual inspection, leading to time-consuming and labor-intensive processes prone to human error. This study presents a novel approach incorporating machine learning techniques to establish a robust fruit shape classification system. We trained and evaluated seven supervised machine learning algorithms by leveraging a public dataset derived from the Tomato Analyzer tool and considering the current four classification systems as label variables. Subsequently, based on class-specific metrics, we derived a novel classification framework comprising seven discernible shape classes. The results demonstrate the superiority of the Support Vector Machine model in terms of its accuracy, surpassing human classifiers across all classification systems. The new classification system achieved the highest accuracy, averaging 88%, and maintained a similar performance when validated with an independent dataset. Positioned as a common standard, this system contributes to standardizing tomato fruit shape classification, enhancing accuracy, and promoting consensus among researchers. Its implementation will serve as a valuable tool for overcoming bias in visual classification, thereby fostering a deeper understanding of consumer preferences and facilitating genetic studies on fruit shape morphometry.

## 1. Introduction

Fruit shape emerges as a critical quality criterion in tomato (*Solanum lycopersicum* L.) production, significantly influencing the preferences of distinct market segments and defining the ultimate destination of the harvest [1,2]. In the fresh market, *ellipsoid*, *round*, *heart*, *flat*, and large tomatoes are favored among consumers [3]. Conversely, *rectangular* and *blocky* shapes dominate the processing tomato industry due to their practical advantages in mechanical harvesting and canning [4]. These shapes are preferred for products like tomato paste, sauce, and canned and diced tomatoes. Additionally, *flat* and large tomatoes are used in fresh markets, as slicing varieties for sandwiches and hamburgers [3]. This difference in market preferences underlines the economic importance of fruit morphology in meeting consumer and industrial requirements.

The cultivated tomato exhibits larger fruits with a much greater shape diversity than its wild relative, which is characterized by round fruits weighing only a few grams. This variation in fruit shape and size occurred in a two-step domestication process. Firstly the tomato’s wild relative was domesticated in northern Peru and then moved to Mesoamerica, where it was finally improved and transformed into the modern tomatoes we know [5,6]. It is proposed that this variation arose early in the domestication process through the selection of alleles with variable shapes and that these alleles accumulated over time, resulting in the modern tomato [7].

Crop breeding is crucial to ensuring future food security, and applying various integrated biological data tools is necessary to maintain continuous improvement. Among the biological data tools currently employed by the scientific community, phenomics enables an understanding of genetic, phenotypic, and environmental relationships. However, obtaining reliable and valuable high-throughput phenotypic information remains a complex task. Currently, plant phenomics applications emphasize high-throughput, non-invasive measurements to provide critical multidimensional data across different organizational levels, developmental stages, and environmental conditions [8,9].

Recent advancements in computer vision methods have revolutionized the agricultural sector, enabling the monitoring of healthy crop growth, control of diseases, pests, and weeds, automatic harvesting, and yield estimation [10]. Additionally, machine vision facilitates phenotyping, supporting downstream genomic selection efforts contributing to increased genetic gains, and improving crop productivity [11,12]. The Tomato Analyzer (TA) is an example of computer vision implementation in tomato crops. This program permits the semi-automated and objective measurement of 47 fruit shape, size, and color descriptors obtained from the longitudinal and latitudinal section of tomato [13,14]. The extensive image datasets generated by TA would be suitable for automatic fruit shape classification. However, the handling and processing of image data is still laborious and time-consuming, which poses a significant obstacle to knowledge generation. Considering these outlined challenges, the use of machine learning techniques appears to be crucial in enhancing the robustness of plant phenotyping methodologies since they offer a promising alternative for the objective and efficient evaluation of plant traits [12,15].

### Machine-Learning Models and Classification Systems

The most prevalent traditional automatic classification algorithms currently encompass both parametric such as Linear and Quadratic Discriminant Analysis (LDA/QDA) [16,17] and Multiple Linear Regression (MLR) [18] and non-parametric models such as Support Vector Machines (SVMs) [19], Artificial Neural Networks (ANNs) [20], Random Forest (RF) [21], and Decision Trees (DTs) [22]. LDA and QDA are probability-based classification methods with high interpretability, especially LDA. Both methods allow for a deeper understanding of the contribution of each phenotypic characteristic, with LDA being more robust to noise and QDA tending to overfit noisy data. MLR, like LDA, assumes linear decision boundaries, offers high interpretability, performs better than QDA in the presence of noise, and provides coefficients that indicate the importance of each phenotypic characteristic. DTs and RF are based on the recursive partitioning of data using information gain. A DT is highly interpretable but prone to overfitting with noise; RF loses interpretability due to its nature, and consensus of multiple trees, which makes it more robust to noise. SVMs and ANNs can capture complex relationships and are practically non-interpretable methods. SVMs aim to find the optimal hyperplane that maximizes the margin among classes in a high-dimensional feature space, effectively separating data points of different categories, is practically non-interpretable, and is quite robust to noise with the appropriate choice of kernel. ANNs process input data through layers of interconnected nodes, adjusting weights and biases to map inputs to desired outputs, mimicking the human brain’s learning process, and can be robust to noise with proper architecture and regularization, but can also overfit.

Although automation is widely adopted in agriculture, automatic fruit shape recognition remains a challenging task. This challenge stems from the difficulty of describing shapes verbally in a detailed and standardized manner, and the variability of shapes under different environmental conditions further complicates this process. Consequently, most crops still rely on visual evaluation for the classification of product shapes. Traditionally, fruit classification is performed by comparing sample patterns defined by agricultural authorities with the actual fruit. However, the criteria for judgment are often not well-defined, can vary among samples, and depend on the subjectivity of the agricultural experts conducting the classification. To improve accuracy, technical expertise is required to understand the varying criteria among different samples [23]. Nonetheless, these subjective evaluations introduce ambiguity and a lack of precision in phenotyping, making it difficult to identify new genes and unravel the complex interactions that determine fruit shape.

**Figure 1:**
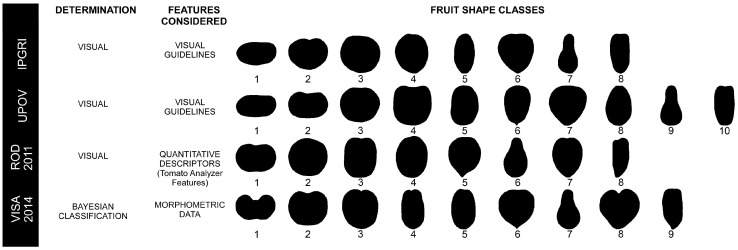
Illustration of a representative fruit of each class for the different shape classification systems available for tomato. Numbers indicate different classes for each shape classification system. (**IPGRI**) 1: *flattened*; 2: *slightly flattened*; 3: *rounded*; 4: *high rounded*; 5: *ellipsoid*; 6: *heart-shaped*; 7: *pyriform*; 8: *cylindrical*. (**UPOV**) 1: *flattened*; 2: *slightly flattened*; 3: *circular*; 4: *rectangular*; 5: *elliptic*; 6: *obovate*; 7: *heart-shaped*; 8: *ovate*; 9: *pear-shaped*; 10: *cylindrical*. (**ROD2011**) 1: *flat*; 2: *round*; 3: *rectangular*; 4: *ellipsoid*; 5: *heart*; 6: *obovoid*; 7: *oxheart*; 8: *long*. (**VISA2014**) 1: *flat*; 2: *round*; 3: *rectangular*; 4: *long-rectangular*; 5: *ellipsoid*; 6: *heart*; 7: *obovoid*; 8: *oxheart*; 9: *long*.

Currently, four systems are available to classify the tomato varieties based on their fruit shape which are detailed in Figure 1. The IPGRI (1996) [24] and UPOV (2001) [25] systems initially established the guidelines using visual descriptors, proposing a total of ten and eight shape classes, respectively. Subsequently, Rodríguez, Muños et al. [26] performed a visual classification of 368 tomato accessions, followed by a refined analysis of a subset of 120 accessions. In this subset, they integrated variables obtained through TA analysis and applied linear discriminant analysis. Through an iterative inclusion of variables, they identified seven principal parameters, yielding an accuracy rate of 83%. Later, Visa et al. (2014) [27] used morphometric data from scanned tomato fruits and elliptic Fourier shape modeling to define the fruit boundaries. They applied a Bayesian classification technique to identify the optimal number of shape categories, computationally and visually identifying nine different tomato shapes. From now on these classification systems will be named: **UPOV**, **IPGRI**, **ROD2011**, and **VISA2014**, respectively. However, the current guidelines for tomato fruit shape classification seem to show discrepancies among the named systems, leading to a lack of consensus among researchers on the most appropriate approach. This observed discrepancy has become noticeable in recent years, with researchers using classification systems proposed by **IPGRI** [28,29,30,31], **UPOV** [32,33], **ROD2011** [4], or their own adapted systems [34,35,36,37] without clearly defined criteria.

The main objective of this study is to develop and validate a machine learning-based system for the automatic classification of tomato fruit shapes, to improve accuracy and reduce subjectivity in the visual characterization process. To achieve this, we evaluated four existing classification systems and seven supervised machine learning algorithms using a public tomato dataset with features extracted from the Tomato Analyzer. We then compared the performance of the automated system with expert visual classification to validate our models. Our preliminary results indicate that the top models achieved over 85% accuracy, outperforming the visual classification. Additionally, we tested the system on an independent dataset to confirm its robustness. This automated system streamlines the classification process, reduces subjectivity, and enhances accuracy, offering a valuable tool for researchers and practitioners in agriculture.

## 2. Materials and Methods

**Figure 2:**
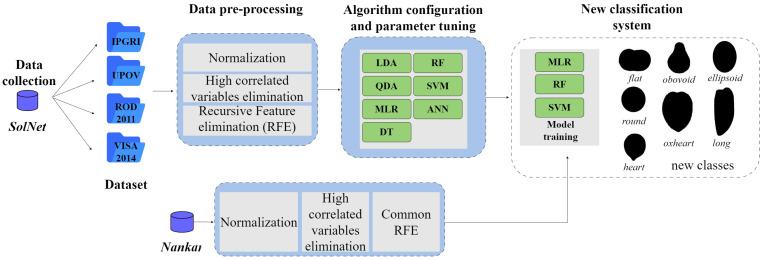
General workflow to define a standardized fruit shape classification system in tomato. Two independent datasets were utilized: *SolNet* and *Nankar*. Four classification systems for fruit shape were considered: IPGRI [24], UPOV [25], ROD2011 [26], and VISA2014 [27]. Seven machine-learning models were analyzed. LDA: Linear Discriminant Analysis; QDA: Quadratic Discriminant Analysis; MLR: Multinomial Logistic Regression; DT: Decision Trees; RF Random Forests; SVM: Support Vector Machines; ANN: Artificial Neural Networks.

The present study analyzes seven widely used machine learning algorithms and four available systems for tomato fruit shape classification. The workflow proposed is represented in Figure 2.

**Datasets**

Two independent datasets were utilized for the analysis.

- *SolNet* dataset: This publicly available dataset from SolGenomics (https://solgenomics.net/, accessed on 8 August 2024) includes 1424 images representing 368 tomato accessions, along with 41 morphological traits and 4 categorical shape features, corresponding to each shape classification system.
- *Nankar* dataset: This dataset contains 145 images of 60 tomato accessions. These images are a subset of the original data from Nankar et al. (2020) [35].

Firstly, the SolNet dataset was implemented for algorithm configuration and parameter tuning to establish the machine-learning models and assess the performance of the classification systems. An initial comparison between automatic and visual classification accuracy revealed limitations in current methods, prompting the development of a novel classification system. This new system was evaluated using the top-performing models and the Nankar dataset.

### 2.1. Dataset Pre-Processing

In the first stage, the *SolNet* dataset, composed of images of longitudinal cuts of tomato fruits, was employed. The original images containing multiple fruits were segmented into individual fruit images, and morphological features were obtained from the original publication using Tomato Analyzer version 2.0 [26]. These images were visually classified into shape categories according to the four available classification systems, by two independent researchers verifying the classifications to ensure accuracy and minimize observational errors.

Descriptive statistics, including minimum, maximum, mean, and standard deviation, were calculated for all attributes within the dataset. The relationship between morphological classes and the fruit features was represented as a boxplot. Normality was assessed using graphical methods, such as histograms and QQ plots [38], alongside the Shapiro–Wilk normality test [39] adjusted by Bonferroni correction [40]. Multivariate normality was evaluated using the MVN package, applying Mardia [41], Henze–Zirkler [42], and Royston [43] tests. Covariance matrix contrasts were further analyzed with the biotools package.

Phenotypic correlations between features were determined using the Spearman test via the rcorr function from the Hmisc package [44]. Principal Component Analysis (PCA) was performed to summarize and visualize the positioning of accessions based on inter-correlated quantitative variables, employing the PCA function from the FactoMineR package [45]. Eigenvalues were analyzed to determine the number of principal components to retain, and the contribution of variables and their correlation with the principal components were evaluated.

The attributes were clustered using the K-means algorithm, with the optimal number of clusters identified by Gap Statistics using the clusGap function from the cluster package [46]. A biplot based on the first two principal components was generated, with accessions colored according to their assigned shape classes and K-means clusters.

Numerical variables were normalized using a z-score approach, and highly correlated variables (correlation coefficient greater than 0.95) were excluded. The dataset was split into training (80%) and testing (20%) sets using the caret package [47]. This function performs a stratified random split, preserving the distribution of the outcome variable and maintaining the representativeness of classes. To enhance model accuracy, Recursive Feature Elimination (RFE) was conducted using the mt package [48], implementing an embedded Support Vector Machine Recursive Feature Elimination (SVM-RFE) procedure. This feature selection process was applied independently for each classification system.

### 2.2. Algorithm Configuration and Parameter Tuning

To optimize the training hyperparameters of the algorithms, we used the mlr package [49] to set up a parameter grid for iterative exploration. The parameter values were selected based on the algorithms used, as summarized in Table 1.

Categorical outcomes were predicted in a test dataset to evaluate the performance of models. This generated a prediction data frame, with numerical outputs replaced by their corresponding categorical labels. A confusion matrix was constructed to calculate the accuracy metric by quantifying the agreement between the predicted and true classes. The overall performance of models was evaluated using standard multi-class classification metrics, including accuracy (Acc), precision (Pr), recall or sensitivity (Rec), and F1 score [50]. Precision, recall, and F1 scores were also examined for each class, and a comprehensive evaluation was conducted. The best-performing models were selected based on overall accuracy.

Hyperparameters were customized and metrics were assessed using the R programming language. Packages such as caret [47], dplyr [51], nnet [52], rpart [53], randomForest [54], ranger [55], e1071 [56], and neuralnet [57] were employed.

### 2.3. Proposal of a New Classification System

To assess and compare the results of the automatic classification against the expert-based classification, we conducted a study using 20 images from the *SolNet* dataset, which were presented in an online survey (https://docs.google.com/forms/d/e/1FAIpQLScD__PD_yVm7sfFfvp8_9m5QUpOsFPAvs3bai1zae6qrmgakg/viewform, accessed on 5 June 2023).

We selected 1 representative image per class and 12 challenging cases from a dataset not previously encountered by the models, ensuring unbiased comparisons.

For computational classification, we used the **ROD2011** system and SVM algorithm, which showed the highest accuracy. The survey images were treated as a test subset with k-fold cross-validation (k = 5). Meanwhile, for visual classification, we polled 34 tomato biology experts who classified the images of tomato shapes by comparing them with the Rodríguez, Muños et al. (2011) [26] guidelines. Images were randomized to minimize bias, and performance metrics were computed using custom code.

We analyzed accuracy, precision, recall, and F1 score for both expert and automated classifications. We used the Kruskal–Wallis test and Dunn test with Bonferroni adjustment for statistical comparisons with the rstatix package [58]. Inter-rater reliability was assessed with the Kappa metric using the irr package [59].

Based on survey feedback, we revised the **ROD2011** classification system by merging *ellipsoid* and *rectangular* classes into a single *ellipsoid* class. Data pre-processing, parameter tuning, model training, and performance evaluation were carried out according to the methods detailed in Section 2.1 and Section 2.2.

### 2.4. Performance of New Classification Systems

To rigorously assess differences in model performance across classification systems, we performed a 5-fold cross-validation and comparative analysis using the MLR, RF, and SVM models, which showed the highest accuracy. We ensured consistency by retaining only the common variables across classification systems identified by RFE. The machine-learning models were trained and tested using the packages mentioned in Section 2.2.

We calculated mean and standard deviation values and assessed homoscedasticity using the Levene test. To evaluate differences in accuracy between automatic and visual classifications, we performed the Wilcoxon–Mann–Whitney test, utilizing the car [60] and dplyr [51] packages in R.

We applied the classification system to an independent dataset, the *Nankar* dataset, for broader validation. This is a subset of the original data from Nankar et al. (2020) [35] which was randomly selected to represent all shape classes while maintaining original frequencies. The dataset underwent pre-processing similar to that in the previous steps, and fruits were classified into the proposed seven shape classes by two independent experts. The MLR, SVM, and RF algorithms were trained on the *SolNet* dataset and tested on the *Nankar* dataset. The common set of variables obtained previously was used in the analysis. Performance was evaluated using accuracy, precision, recall, and F1 score, as detailed in Section 2.2.

## 3. Results

### 3.1. Dataset Pre-Processing

The dataset exhibited considerable variability in most traits, with coefficients of variation ranging from 4.6% for “Distal Eccentricity” to 375% for “Shoulder Height”. High values for the interquartile range and the range between minimum and maximum indicate substantial diversity in traits (see Table S1). The *SolNet* dataset was representative of the fruit shape classes across different classification systems. The most frequent categories were *ellipsoid* (26.1% in **ROD2011** and 26.2% in **VISA2014**), *elliptic* (20.3% in **UPOV**), and *high rounded* (18.8% in **IPGRI**). Conversely, less common classes included *oxheart* (2.7% in **ROD2011** and **VISA2014**), *obovate* (1.4% in **UPOV**), and *heart-shaped* (8.1% in **IPGRI**).

**Figure 3:**
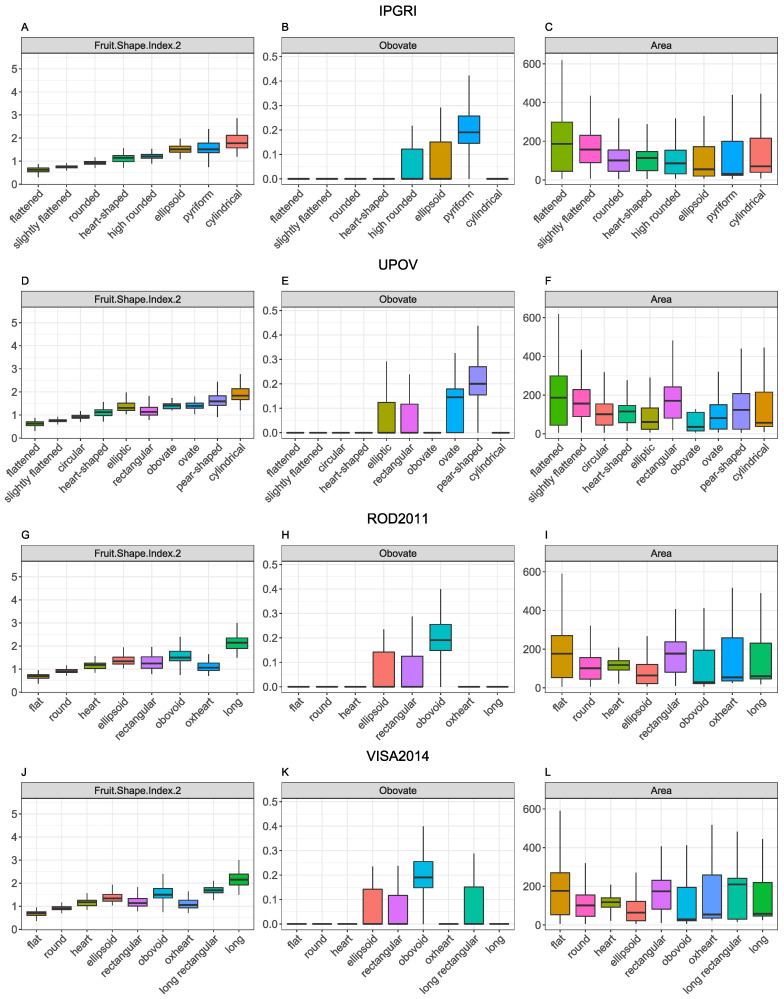
Box plots representing the value of shape traits across morphological classes in each classification system. The middle line of the box indicates the median of the data, while the top and bottom ends of the box indicate the 25th and 75th percentiles. The length of the box is the difference between these two percentiles and is known as the interquartile range (IQR). The whiskers represent the expected variance of the data. The box plot displays whiskers that extend 1.5 times the IQR from the top and bottom ends. (**A**–**C**) **IPGRI** classification system. (**D**–**F**) **UPOV** classification system. (**G**–**I**) **ROD2011** classification system. (**J**–**L**) **VISA2014** classification system. Different colors denote different classes in each classification system. The color scale is located to the left of the plots.

Box plots were utilized to elucidate the relationship between morphological classes and traits within each classification system. Notably, some features showed distinct patterns between shape classes, such as lower values for “Fruit.Shape.Index.2” in *flattened* shapes and higher values in elongated shapes like *long*, *long-rectangular*, *obovoid*, *pyriform*, and *cylindrical* (Figure 3A,D,G,J). However, class differentiation by traits like “Area” (Figure 3C,F,I,L) was challenging due to significant overlap and dispersion. By contrast, some traits, such as “Obovate” (Figure 3B,E,H,K), allowed distinguished specific morphological classes to be formed.

The analysis of distribution revealed that most traits did not follow a normal distribution. Multivariate normality tests indicated significant deviations from multivariate normality (see Table S3, Figure S1). The contrast analysis of covariance matrices showed non-uniform covariance matrices among classes, with 91.22% of correlations being significant. Within this group, 2.44% had correlations greater than 0.85, and 5.98% had moderate correlations ranging from 0.60 to 0.85 (see Table S4, Figure S2).

The PCA demonstrated that the first two principal components explained 44.5% of the variance. Visualization of fruit by shape classes along these components revealed overlapping patterns among classes (see Figure S3). The traits were grouped into eight clusters by k-means clustering, highlighting patterns and relationships that could contribute to data variability (see Figure S4). This suggests the potential for dimensionality reduction in subsequent analyses.

Four highly correlated variables were excluded from the analysis: “Width.Mid.height”, “Height.Mid.width”, “Fruit.Shape.Index.1”, and “Perimeter”. The dataset was split into training (1142 images) and test subsets (282 images). It is worth noting that the subsets, like the overall dataset, exhibited class imbalances, particularly with minority classes such as *oxheart* in **ROD2011** and **VISA2014** (2.7%), and *obovate* in **UPOV** (1.4%). In contrast, the **IPGRI** subset had a balanced representation across classes. Detailed frequency information for each category is provided in Table S2.

The RFE method performed distinctive variable feature selections across different classification systems. However, a consensus emerged regarding the primary ranked variables, with “Fruit.Shape.Index.2” consistently identified as the highest-ranked feature across all datasets analyzed. The number of selected features varied by classification system, with **ROD2011** and **VISA2014** selecting 18 traits each, **UPOV** selecting 28 traits, and **IPGRI** selecting 26 traits. Information on ranked features and selected subsets is detailed in Table S5.

### 3.2. Algorithm Configuration and Parameter Tuning

Table 2 presents a summary of the accuracy, precision, recall, and F1 score results obtained from evaluating the seven models across the different classification systems.

Considering the overall accuracy across all of the classification systems, the QDA model consistently showed a lower accuracy compared to LDA and DT, with the lowest performance observed particularly on the **UPOV** system. In contrast, the MLR, SVM, and RF algorithms demonstrated higher accuracies, with RF achieving the highest accuracy of 84.40% on the **ROD2011** dataset. The ANN models exhibited major differences between training and testing, showing a strong performance in training but less effectiveness in testing.

Furthermore, performance varied by the classification system. The **UPOV** system generally had the lowest accuracy across most models, except for RF. In contrast, the **IPGRI** and **VISA2014** systems had intermediate accuracy values, while **ROD2011** showed the highest accuracy, except where **VISA2014** outperformed **ROD2011** in the LDA model.

The class-specific analysis underscored the challenges in classification across certain classes for all models (see Table S6). This detailed analysis of class-specific performance revealed both strengths and weaknesses in classification, with certain shapes posing consistent challenges across models. Across the classification systems, the flattened and rounded shapes generally demonstrated the best performances, achieving high accuracy and F1 scores. Conversely, the rectangular and heart- shapes exhibited poor performance across most models.

The **UPOV** system faced significant difficulties with the *obovate* and *ovate* classes, particularly with the DT model, and the **rectangular** shape also underperformed. The **IPGRI** system showed better results for the *rounded* and *pyriform* shapes but struggled with *slightly flattened* and *ellipsoid* shapes. The **ROD2011** system encountered challenges in accurately classifying the *oxheart* and *rectangular* shapes, meanwhile, the *flat* shape showed strong performance. Similarly, the **VISA2014** system displayed robust performance for the *flat* shape but had issues with the *rectangular* and *oxheart* shapes.

### 3.3. Proposal of a New Classification System

A survey with images representing all the fruit shape classes, including five *ellipsoid*, two *flat*, two *heart-shaped*, four *long*, three *obovoid*, two *oxheart*, one *rectangular*, and one *round*, was distributed among tomato experts for visual classification.

Expert visual classification resulted in a mean accuracy of 0.56 with a standard deviation of 9%. The high standard deviation reflected the variability among experts, confirmed by the inter-rater reliability test, which yielded a kappa value of −0.03, indicating less agreement than expected by chance. In contrast, automatic classification achieved a mean accuracy of 0.70 with a 4% standard deviation. A statistically significant difference between expert-based and automatic classification was found (*p* < 0.001).

The performance metrics revealed that classes such as *flat*, *long*, and *round* had the highest F1 scores in expert classification (0.81, 0.76, and 0.73, respectively). However, the *oxheart* class had the lowest performance metrics, and the *rectangular* class showed a low precision but high recall, indicating that fruits belonging to another class, such as *ellipsoid* and *round*, were classified as *rectangular*. The automatic classification outperformed the expert-based classification in most classes, except for the *long* class. Notably, the *flat* and *round* classes performed well in both systems, with F1 scores of 0.92 and 0.87, respectively. However, the *oxheart* class only achieved an F1 score of 0.47 (see Table S7).

Based on the observed difficulties in distinguishing *ellipsoid* and *rectangular* shapes, these classes were merged into a single *ellipsoid* class. Using Recursive Feature Elimination (RFE), 16 variables were selected and distributed across seven of the eight clusters identified in the previous K-means Cluster Analysis (Section 3.2). The top five ranked traits in RFE were “Fruit.Shape.Index.2”, “Internal.Fruit.Shape.Index”, “Distal.Angle.Macro” (20%), and “Proximal.Angle.Macro” (10% and 20%), which align with the traits identified in **ROD2011**.

**Figure 4:**
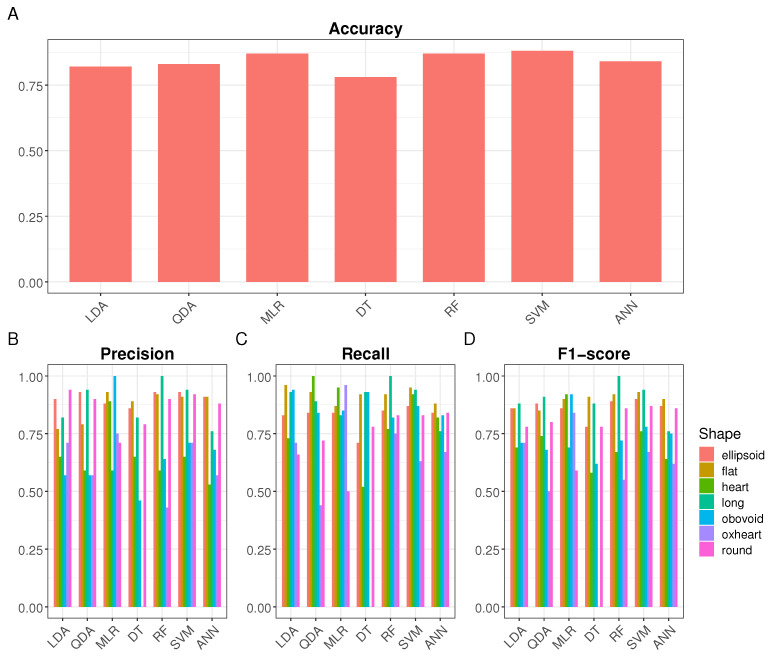
Values for performance metrics of individual classes in the new classification system proposed. (**A**) Accuracy value for each machine-learning algorithm. (**B**) Precision values. (**C**) Recall values. (**D**) F1 score values. Different colors represent the shape classes. LDA: Linear Discriminant Analysis; QDA: Quadratic Discriminant Analysis; MLR: Multinomial Logistic Regression; DT: Decision Trees; RF Random Forests; SVM: Support Vector Machines; ANN: Artificial Neural Networks.

The model accuracy ranged from 0.78 for Decision Trees (DT) to 0.88 for Support Vector Machines (SVM) (see Figure 4A). Accuracy improved across all models with the new classification system, demonstrating that removing the *rectangular* class enhanced overall classification effectiveness.

When examining class-specific performance metrics (see Figure 4B–D), some challenges were encountered by models in classifying different classes. Across various models, certain classes stood out with high F1 scores, such as the *long* class in the LDA, QDA, RF, and SVM models, and the *heart* and *obovoid* classes in the MLR model. Conversely, some classes posed significant challenges, such as the *oxheart* class across multiple models and the *heart* class in the LDA model. Additionally, specific models struggled with particular classes, like the *round* class in the MLR model. Overall, these findings underscore the varied performance of models in classifying different classes, with some classes being more challenging to classify accurately than others.

### 3.4. Performance of New Classification Systems

From the previous variables selected by RFE, a subset of 12 variables was consistently identified in all datasets. These variables included: “Fruit.Shape.Index.2”, “Distal.Angle.Macro” (10 and 20%), “Proximal.Angle.Macro” (10 and 20%), “Proximal.Angle.Micro” (5%), “Circular”, and “Elliptic”, “Proximal.Fruit.Blockiness” (20%), “Distal.Fruit.Blockiness” (5%), “Rectangular”, and “Internal.Fruit.Shape.Index”. These selected traits aligned with five of the eight clusters derived through the k-means cluster analysis. The variable clusters are summarized as follows: Cluster 1, characterized by “Fruit Shape Index” (2), “Distal.Angle.Macro” (10 and 20%), and “Proximal.Angle.Macro” (20%); Cluster 2, represented by *circular* and *elliptic*; Cluster 3, featuring “Proximal.Fruit.Blockiness” (20%); Cluster 7, which included “rectangular” and “Internal.Fruit.Shape.Index”; and Cluster 8, encompassing “Proximal.Angle.Micro” (5%), “Proximal.Angle.Macro” (10%), and “Distal.Fruit.Blockiness” (5%).

**Figure 5:**
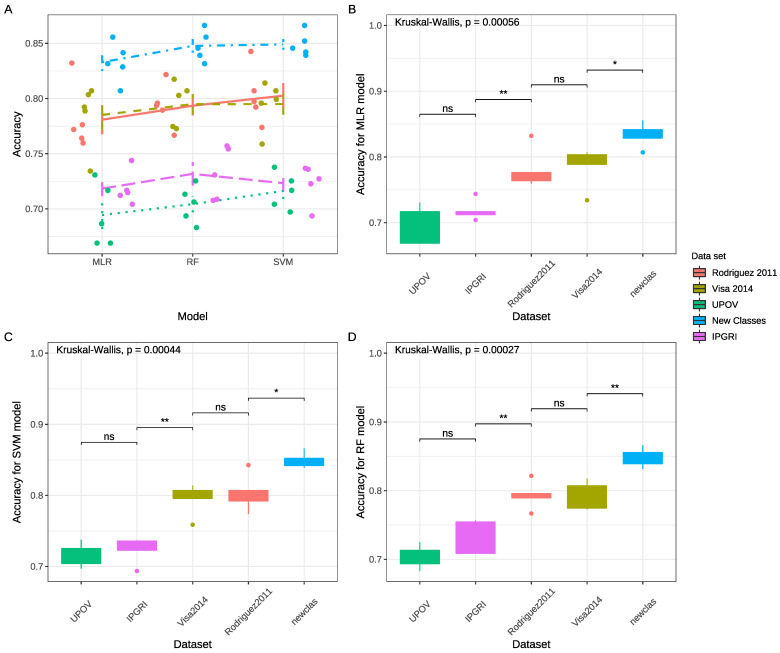
Comparison of best-performing models for 5-fold cross-validation. (**A**) Mean accuracy and standard deviation for Support Vector Machine (SVM), Random Forest (RF), and Multinomial Logistic Regression (MLR) models. Dots represent the mean value for each 5-fold cross-validation. (**B**–**D**) Box plot of accuracy for different models. The middle line of the box indicates the median of the data, while the top and bottom ends of the box indicate the 25th and 75th percentiles. The whiskers represent the expected variance of the data. Dots show the outliers’ values. Different colors denote the shape classification systems. The color scale is located to the left of the plots [14,27]. Wilcoxon comparison significance: ns: *p* > 0.05; *: *p* ≤ 0.05; **: *p* ≤ 0.01.

In our study, the mean accuracy values considering the models ranged from 0.69 to 0.85, with standard deviations between 0.01 and 0.03 (Figure 5A). The MLR model applied to the **UPOV** dataset showed the lowest accuracy, while the SVM model with the new set of classes achieved the highest mean accuracy.

No significant differences in mean accuracy were observed across models at a 5% significance level, although differences were significant among classification systems (*p* < 0.01) (Figure 5B–D). The Wilcoxon–Mann–Whitney test revealed no significant difference in mean accuracy between the **UPOV** and **IPGRI** datasets, both of which displayed the lowest accuracy. In contrast, the **ROD2011** and **VISA2014** datasets showed intermediate accuracy values and no significant difference between them, with the novel classification system yielding the highest accuracy across all models.

For a broader validation of the novel classification system, the top-performing models were evaluated using the *Nankar* dataset. The distribution of tomato fruit shapes in this dataset revealed a predominance of *flat*, *ellipsoid*, and *round* classes, which together represent 66.9.

The RF model achieved the highest overall accuracy at 87.59%, followed by the SVM model at 86.90%, and the MLR model at 82.76%. These results align with those presented in Section 3.3, where the new classification system was proposed, indicating a maximum of 25 misclassified images.

In terms of precision, the RF, SVM, and MLR models scored 0.87, 0.86, and 0.82, respectively. The recall values were 0.82 for the SVM model, 0.82 for the RF model, and 0.78 for the MLR model. The F1 scores were 0.83, 0.82, and 0.79 for the RF, SVM, and MLR models, respectively. The lower recall and F1 scores for the MLR model indicate a tendency to miss true positive cases, resulting in more false negatives and, consequently, a lower overall performance (see Table 3).

**Figure 6:**
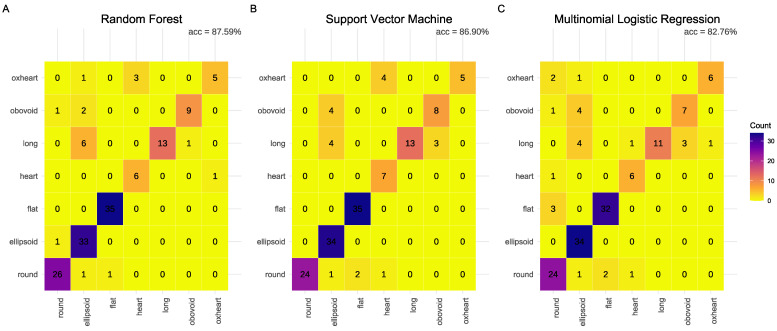
Confusion matrix summarizing the performance of best-performing models in the *Nankar* dataset in the new shape classification system. (**A**) Random Forest model. (**B**) Support Vector Machine. (**C**) Multinomial Logistic Regression model. The rows represent the true classes, while the columns represent the predicted classes. The diagonal denotes the labels that were correctly classified.

Considering the class-specific metrics, the *flat* class achieved the highest F1 score across all models. In contrast, the RF model recorded the lowest F1 score for the *oxheart* class, with a value of 0.67. Most of the misclassified *oxheart* fruits were incorrectly assigned to the *heart* class in this model (Figure 6A). The SVM and MLR models mainly failed to detect *obovoid* shapes, yielding F1 scores of 0.70 and 0.64, respectively. These misclassified fruits were predominantly assigned to the *ellipsoid* class, as illustrated in Figure 6B,C.

## 4. Discussion

### 4.1. Comparison of Existing Classification Systems and Performance of Machine Learning Models

Fruit shape is one of the most important quality aspects for tomatoes, defining not only the consumer preference but also relevant aspects of the marketing demand and exportation requirements. A description of an agricultural product’s shape is often necessary to investigate the heritability of fruit shape descriptors for cultivar descriptions, variety registration (for intellectual property rights), and the evaluation of consumer decision performance. Despite these, to date, tomato-shape grading has mainly been based on visual inspection, which is highly subjective, time-consuming, and labor-intensive [61,62].

Recent studies have shown that combining image-based phenotyping with machine learning techniques can lead to robust and accurate recognition and classification in various crops [63,64,65,66]. In this study, we utilized fruit shape attribute data obtained from images of longitudinal cut fruit sections using the Tomato Analyzer application. The TA data, combined with supervised machine learning algorithms provided a classification approach that accurately assigned fruits to define the shape classes, surpassing visual inspection made by the experts. The complete approach was performed on the four available classification systems and a new system was proposed. By comparing the mean of the models, the best scheme was defined as a common standard for tomato shape classification, which was validated on an independent dataset. Therefore, this approach provides a standard for the classification of tomato fruits and could be replicated for other vegetables.

At present, there exist four principal systems for the classification of fruit shapes in tomato. Nonetheless, the existing guidelines exhibit inadequacies, leading to a lack of agreement among researchers who use them without well-defined guidelines. Consequently, it is essential to create a controlled and objective classification system that can gain widespread acceptance within the research community. Our analysis has revealed that the **UPOV** and **IPGRI** classification systems demonstrate lower overall accuracy values across all models. Conversely, the **ROD2011** and **VISA2014** systems are the superior performers. In a comparative analysis among the three top-performing models (MLR, SVM, and RF), the **UPOV** and **IPGRI** systems showed no significant divergence but differed from the **ROD2011** and **VISA2014** systems, which in turn exhibited no discernible differences between each other. These variations in mean accuracy may be attributed to the fact that the **UPOV** and **IPGRI** systems rely on visual assessment, which can introduce bias in categorization. Additionally, these classification systems exhibit inconsistent criteria, categories, and fluctuating terminology regarding fruit shapes. Moreover, some terms used lack consistency with prevailing ontological standards. Meanwhile, the system proposed by Rodríguez, Muños et al. (2011) [26] incorporates the analysis of TA features, which are numeric and objective data. The work of Visa et al. (2014) [27] builds upon the previous work of Rodríguez, Muños et al. (2011) [26] but also uses morphometric data for computational classification.

### 4.2. Challenges in Class-Specific Classification

The classification of fruits and vegetables poses a great challenge due to their inherent diversity and complexity, resulting in inter- and intra-class variations [67]. The analysis of the *SolNet* dataset, which is representative of tomato germplasm, revealed the capability of certain Tomato Analyzer traits to distinguish patterns among shape classes. The PCs and k-means cluster analyses suggested the potential for dimensionality reduction, grouping the 41 analyzed traits into eight clusters. The RFE analysis resulted in distinct rankings for traits across classification systems. Nevertheless, the “Fruit Shape Index”, which relates the height and width of fruits and gives a general idea of the shape, consistently emerged as the most significant trait in shape variation explanation. Across all systems 12 main traits were selected, which reflected five of the previously identified clusters. These findings align with Rodríguez, Muños et al. (2011) [26], who identified the “Fruit Shape Index” as the main feature defining grading fruit morphology.

As accuracy is the most widely used metric for classifiers [68], we focus on this estimator as the selection criterion. Noteworthy LDA, QDA, and DT consistently emerged as the worst-performing models across all classification systems. Conversely, MLR, RF, and SVM showed superior performance. Notably, the ANN model showed an outstanding performance on the training dataset, but its accuracy significantly dropped on the test dataset. In addition, challenges were encountered in accurately classifying certain shapes. In particular, *slightly flattened* and *obovate* shapes in the **IPGRI** and **UPOV** systems, respectively, and the *oxheart* class showed the lowest overall F1 scores in **ROD2011**, **VISA2014**, and the new systems across all models.

Discrepancies among models and challenges in class-specific classification may be partly due to the sensitivity of algorithms to class imbalance and overlap in datasets [69,70]. This hypothesis is supported by the high correspondence between higher error rates and lower overall predictive performance with the under-represented classes, emphasizing the critical importance of addressing class imbalances. Various approaches, such as oversampling, undersampling, boosting, bagging, and repeated random sub-sampling, can be used to address data imbalances, each with its limitations [71]. Additionally, the size of the dataset has a significant impact on the model’s performance. Traditional machine learning models, such as SVM, have been seen to have more classification advantages on small datasets than deep learning models [72]. This underscores the importance of considering the dataset as well as model characteristics when dealing with imbalanced data scenarios.

### 4.3. Proposal of a New Classification System

A comparative analysis between visual and automated tomato shape classification showed that the automated method, taking into account the SVM algorithm and the **ROD2011** system consistently outperformed the visual method. Performance metrics revealed challenges in classifying certain shapes, particularly the *oxheart* and *rectangular* classes, highlighting the need for further refinement. In the survey, experts often classified the *rectangular* and *ellipsoid* fruits interchangeably, leading to an increase in false positives and decreased precision. Genetic studies have shown that similar genes control the fruit shape of *rectangular* and *ellipsoid* fruits [4,62,73,74]. This evidence encouraged us to merge the two classes into a single category named *ellipsoid*.

A novel classification system was developed based on the **ROD2011** fruit classification and the merging of *rectangular* and *ellipsoid* classes. The best-performing machine learning models, MLR, RF, and SVM, were evaluated across all five datasets including the new system. The new classification system resulted in higher mean accuracy values for all models, and the SVM model achieved the highest accuracy, reaching 88% and 87% on two independent datasets of *SolNet* and *Nankar*, respectively. Based on the comparative findings between existing classification systems and the results observed in this study, we believe that this system will serve as a common standard for tomato fruit shape classification. This novel approach not only improves the accuracy of tomato cultivar delineation but also promotes consensus among researchers.

## 5. Conclusions

This research outlines a comprehensive approach to developing an automated and objective fruit shape classification system for tomatoes using advanced technologies like computer vision and machine learning. Evaluating seven supervised learning algorithms and four classification systems, SVM emerged as the most effective model, surpassing visual classification by experts with varying agreement levels. By refining Rodríguez, Muños et al.’s (2011) [26] system and eliminating the redundant rectangular class, our approach achieved an approximately 88% accuracy, validated on an independent dataset for reliability. This positions our method as a standard for tomato fruit shape classification, significantly advancing automated horticultural practices. It represents a substantial contribution to investigations into fruit morphology, as well as the accurate description and registration of crop varieties. Future research may extend this approach to other crops and refine necessary model aspects, such as the management of unbalanced data, to enhance accuracy and adaptability.

## Figures and Tables

**Table 1:** Tuning parameters in different supervised classification models.

Algorithm	Parameters	IPGRI	UPOV	ROD2011	VISA2014
LDA		Default	Default	Default	Default
QDA		Default	Default	Default	Default
MLR		Default	Default	Default	Default
DT	max_depth ^1^	5	18	9	10
	cp ^2^	0.001	0.012	0.001	0.001
	min_split ^3^	23	18	13	7
	mtry ^4^	6	8	8	6
RF	num_tree ^5^	300	300	300	300
	node_size^6^	2	1	1	1
	sample_size ^7^	0.80	0.63	0.70	0.80
SVM	C ^8^	5.34	2.16	5.34	2.63
	Gamma ^9^	0.414	4.160	0.414	0.891
	Degree ^10^	5	4	5	7
	kernel ^11^	linear, radial, polynomial	linear, radial, polynomial	linear, radial, polynomial	linear, radial, polynomial
ANN	n_hidden ^12^	3	2	3	3
	n_neurons ^13^	22, 18, 14	25, 17	14, 12, 10	14, 12, 10

LDA: Linear Discriminant Analysis; QDA: Quadratic Discriminant Analysis; MLR: Multinomial Logistic Regression; DT: Decision Trees; RF Random Forests; SVM: Support Vector Machines; ANN: Artificial Neural Networks. ^1^ max_depth: maximum depth in decision trees; ^2^ cp: threshold determining the worthiness of splitting a node; ^3^ min_split: minimum split in a node for a split to be attempted; ^4^ mtry: number of variables considered for splitting at each node; ^5^ num_tree: number of trees in the forest; ^6^ node_size: minimum size of terminal nodes; ^7^ sample_size: proportion of the dataset used for training each tree; ^8^ C: cost parameter which indicates the tolerance for violations of the margin and hyperplane; ^9^ Gamma: represents the inverse of the radius of influence of support vectors; ^10^ Degree: controls the flexibility of the decision boundary used to separate different classes; ^11^ kernel: kernel type; ^12^ n_hidden: number of hidden layers; ^13^ n_neurons: number of neurons in each layer.

**Table 2:** Overall values for performance metrics across distinct classification systems. Accuracy (Acc), precision (Pr), recall (Rec) and F1 score (F1).

Algorithm	IPGRI				UPOV				ROD2011				VISA2014			
	**Pr**	**Rec**	**F1**	**Acc**	**Pr**	**Rec**	**F1**	**Acc**	**Pr**	**Rec**	**F1**	**Acc**	**Pr**	**Rec**	**F1**	**Acc**
LDA	0.69	0.73	0.70	0.70	0.65	0.68	0.66	0.69	0.64	0.77	0.69	0.74	0.69	0.76	0.70	0.75
QDA	0.65	0.67	0.65	0.65	0.65	0.68	0.66	0.64	0.63	0.76	0.67	0.74	0.63	0.70	0.65	0.74
MLR	0.72	0.73	0.72	0.72	0.65	0.65	0.65	0.69	0.74	0.75	0.75	0.82	0.72	0.73	0.72	0.78
DT	0.64	0.67	0.64	0.66	0.54	0.62	0.60	0.65	0.67	0.70	0.68	0.76	0.55	0.54	0.70	0.72
RF	0.75	0.77	0.75	0.76	0.70	0.79	0.72	0.77	0.76	0.81	0.78	0.84	0.66	0.79	0.68	0.80
SVM	0.73	0.75	0.74	0.74	0.66	0.76	0.68	0.73	0.75	0.82	0.77	0.84	0.71	0.86	0.75	0.82
ANN	0.70	0.72	0.71	0.71	0.63	0.62	0.62	0.66	0.69	0.70	0.69	0.78	0.63	0.73	0.64	0.77

LDA: Linear Discriminant Analysis; QDA: Quadratic Discriminant Analysis; MLR: Multinomial Logistic Regression; DT: Decision Trees; RF Random Forests; SVM: Support Vector Machines; ANN: Artificial Neural Networks.

**Table 3:** Values for performance metrics (accuracy, precision, recall, and F1 score) of individual classes in *Nankar* dataset.

Algorithm	*ellipsoid*			*flat*			*heart*			*long*			*obovoid*			*oxheart*			*round*		
	**Pr**	**Rec**	**F1**	**Pr**	**Rec**	**F1**	**Pr**	**Rec**	**F1**	**Pr**	**Rec**	**F1**	**Pr**	**Rec**	**F1**	**Pr**	**Rec**	**F1**	**Pr**	**Rec**	**F1**
MLR	0.77	0.86	0.81	0.77	1.00	0.87	0.94	0.91	0.93	0.75	0.86	0.80	1.00	0.55	0.71	0.70	0.58	0.64	0.86	0.67	0.75
RF	0.93	0.93	0.93	0.77	0.97	0.86	0.97	1.00	0.99	0.67	0.86	0.75	1.00	0.65	0.79	0.90	0.75	0.82	0.83	0.56	0.67
SVM	1.00	0.86	0.92	0.79	1.00	0.88	0.95	1.00	0.97	0.58	1.00	0.74	1.00	0.65	0.79	0.73	0.67	0.70	1.00	0.56	0.71

Precision (Pr), recall (Rec), and F1 score (F1). Multinomial Logistic Regression (MLR), Random Forests (RF), and Support Vector Machines (SVM). Values for accuracy were equal to 0.83, 0.88, and 0.87 for MLR, RF, and SVM algorithms, respectively.

## Data Availability

The SolNet dataset is available on the Consejo Nacional de Investigaciones Científicas y Técnicas, Argentina. Repository https:\\ri.conicet.gov.ar\handle\11336\231857#anchorMain, accessed on 22 August 2024.

## Supplementary Materials

The following supporting information can be downloaded at: https://www.mdpi.com/article/10.3390/plants13172357/s1, Figure S1: Frequency distribution histograms for all the Tomato Analyzer features analyzed.; Figure S2: Correlation matrix representing Spearman correlation coefficients between all the Tomato Analyzer features. The size and colors of circles indicate the correlation of Spearman coefficients (positive or negative) between pairs of traits. Values of upper triangle, diagonal, and correlation coefficients non-significative and above 0.6 are removed from the plot.; Figure S3: Principal component analysis (PCA) of tomato accessions based on Tomato Analyzer features. The biplot the traits and tomato accessions across the first two PCs. The traits are represented as grey arrows. The direction and length of the arrows indicate the weight and sign of the original variables in the two first PCs. Ellipses represent the tomato accessions clustered by shape classes. Different colors and shapes denote the tomato shape classes. (**A**) **IPGRI** classification system. (**B**) **UPOV** classification system. (**C**) **ROD2011** classification system. (**D**) **VISA2014** classification system.; Figure S4: Visualization of variables across the first two PCs, grouped by k-means clusters. The biplot shows the traits colored by k-means clusters. Grey dots represent tomato accessions. The direction and length of the arrows indicate the weight and sign of the original variables in the first two PCs. The distance between arrows indicates the correlations between traits. The arrow colors represent different k-mean clusters. The color scale is located at the bottom of the plots.; Figure S5: Histogram of the existing shape classes in the *Nankar* dataset. Within each box the percentage of that class within the dataset is expressed; Table S1: Descriptive statistics of all the Tomato Analyzer traits; Table S2: Frequencies of shape classes across the different classification systems; Table S3: Normality analysis for all Tomato Analyzer traits; Table S4: Correlation matrix representing Spearman correlation coefficients between all the Tomato Analyzer features; Table S5: Traits selected and ranked by Recursive Feature Selection in each dataset; Table S6: Values for performance metrics (Precision, Recall and F1 score) of individual classes in different classification systems; Table S7: Values for performance metrics (Precision, Recall and F1 score) of individual classes in automatic (Support Vector Machine) and visual classifications.
